# Infant Effortful Control Mediates Relations Between Nondirective Parenting and Internalising-Related Child Behaviours in an Autism-Enriched Infant Cohort

**DOI:** 10.1007/s10803-021-05219-x

**Published:** 2021-08-26

**Authors:** C. G. Smith, E. J. H. Jones, S. V. Wass, G. Pasco, M. H. Johnson, T. Charman, M. W. Wan, The BASIS Team, Simon Baron-Cohen, Anna Blasi, Patrick Bolton, Susie Chandler, Celestee Cheung, Kim Davies, Mayada Elsabbagh, Janice Fernandes, Isabel Gammer, Holly Garwood, Teodora Gliga, Jonathan Green, Jeanne Guiraud, Kristelle Hudry, Michelle Liew, Sarah Lloyd-Fox, Helen Maris, Louise O’Hara, Andrew Pickles, Helena Ribeiro, Erica Salomone, Leslie Tucker, Agnes Volein, Ming Wai Wan

**Affiliations:** 1 Henry Wellcome Building, Institute of Psychiatry, Psychology and Neuroscience, King’s College London, De Crespigny Park, London, SE5 8AF UK; 2 Centre for Brain and Cognitive Development, Birkbeck, University of London, London, UK; 3 School of Psychology, University of East London, London, UK; 4 School of Health Sciences, University of Manchester, Manchester, UK; 5 Department of Psychology, University of Cambridge, Cambridge, UK

**Keywords:** ASD, Internalising, Anxiety, Parent-infant interaction, Temperament, Effortful control, Behavioural inhibition, Infant sibling study

## Abstract

Internalising problems are common within Autism Spectrum Disorder (ASD); early intervention to support those with emerging signs may be warranted. One promising signal lies in how individual differences in temperament are shaped by parenting. Our longitudinal study of infants with and without an older sibling with ASD investigated how parenting associates with infant behavioural inhibition (8–14 months) and later effortful control (24 months) in relation to 3-year internalising symptoms. Mediation analyses suggest nondirective parenting (8 months) was related to fewer internalising problems through an increase in effortful control. Parenting did not moderate the stable predictive relation of behavioural inhibition on later internalising. We discuss the potential for parenting to strengthen protective factors against internalising in infants from an ASD-enriched cohort.

**Supplementary Information:**

The online version contains supplementary material available at 10.1007/s10803-021-05219-x.

## Introduction

Autism Spectrum Disorder (ASD) is a neurodevelopmental condition associated with two core symptom domains: social interaction and communication difficulties, and restricted and repetitive behaviours in tandem with sensory processing atypicalities (DSM-5; American Psychiatric Association, 2013). These core symptoms are often accompanied by additional mental health conditions (Salazar et al., 2015; Simonoff et al., 2008); in particular, internalising-related disorders such as anxiety (van Steensel et al., 2011).

Internalising disorders—in particular, anxiety—affect approximately 40% of individuals with ASD, and are often clinically identified in mid-childhood (Davis et al., 2011). ‘Internalising’ is a broad dimension of psychopathological variation comprising anxiety and mood disturbances, and is commonly used to indicate prodromal symptoms of affective disorders (Kostyrka-Allchorne et al., 2020; Krueger & Markon, 2006; Rueter et al., 1999). The co-occurrence of internalising symptoms and ASD is thought to interact to amplify core difficulties; for example, difficulties in social interaction can increase for those with ASD and anxiety difficulties, as contexts involving social evaluation trigger both anxious and autistic symptoms (Chang et al., 2012). As such, investigation into internalising-related distress within ASD has been identified as a research priority of the autism community (Lord et al., 2020).

Controversy remains about the co-occurrence of internalising disorders and ASD, with varying interpretations available: (a) internalising constitutes a part of ASD; (b) ASD symptoms cause internalising disorders, or (c) internalising disorders and ASD are phenotypically distinct but overlap with regard to early risk factors (Kerns & Kendall, 2012; Wood & Gadow, 2010). Research into the overlapping risk factors for internalising disorders and ASD has increased in recent years (Yarger & Redcay, 2020). To investigate internalising within ASD in early development necessarily involves prospective study of infant cohorts, before the emergence of ASD. The advantages of investigating internalising within ASD from an early developmental perspective are twofold. Early prediction of risk for internalising within ASD could eventually enable intervention that may attenuate emerging affective disorders, reducing the potential for positive feedback between overlapping symptoms and thus having cascading benefits for individuals with ASD. Further, identifying early markers of internalising disorders in infants, before ASD emerges, could help us understand the aetiology of the two conditions’ concurrence.

## Infant Temperamental Predictors of Subsequent Internalising Disorders

Temperament, emerging early in life and defined broadly as ‘the extent to which individuals respond to their environment, and their ability to modulate and control these responses’ is thought to be an early marker for later psychopathology (Kostyrka-Allchorne et al., 2019, p. 401). Several prospective studies of infants with a family history of ASD have investigated temperament, showing early differences in domains such as surgency (indexing active, approach behaviours and positive affect) and effortful control (indexing self-regulatory processes) in infants with a family history of ASD (Clifford et al., 2013) and in those who later develop ASD (Pijl et al., 2019). The extent to which these differences relate to later core symptoms of ASD or whether they could instead relate to co-occurring internalising problems remains largely unclear. However, two recent studies of infant siblings of children with ASD—investigating the same sample as the present study—have examined temperament associations to later ASD symptoms versus the internalising-related symptoms of anxiety. Behavioural inhibition and effortful control were shown to correlate with anxiety and ASD symptoms (Ersoy et al., 2020), while other differences, such as activity levels and inhibitory control, were not (Shephard et al., 2018). This work suggests these two former temperament domains may be particularly important for explaining the development of internalising disorders such as anxiety: (1) behavioural inhibition, and (2) effortful control.

### Behavioural Inhibition

In normative populations, behaviourally inhibited temperament has been shown to predict later childhood internalising problems; in particular, anxiety (Muris et al., 2011; Murray et al., 2009). Early behavioural inhibition is defined as ‘a tendency of some children to withdraw and/or exhibit negative affect in response to novel stimuli (people, places, events, and objects)’ (Gartstein et al., 2010, p. 652) and is broadly characterised as a form of avoidance and distress towards novelty (Fox et al., 2020). These behaviours emerge early, and individual differences are stable from 4 months (Rothbart, 1988; Schmidt et al., 2020). Although not true of all children with a history of behavioural inhibition, those displaying the temperament in infancy are at elevated likelihood of developing anxiety in adulthood (Frenkel et al., 2015). In addition, studies of children with community-referred ASD symptoms show that infants who have inhibited temperaments (as well as other temperament domains, such as negative emotionality) are more likely to have co-occurring internalising symptoms compared to infants with stronger self-regulatory capacities (Chetcuti, et al., 2020a, 2020b; Chetcuti, et al., 2020a, 2020b).

### Effortful Control

Self-regulatory temperamental traits in infancy, such as effortful control, are also thought to relate to later internalising problems, such as anxiety. Effortful control reflects an individual’s ability to activate or inhibit responses and voluntarily control attention (Rothbart et al., 2003) and has a long developmental timecourse that becomes clear over the second year of life (Putnam et al., 2001). In normative populations, reduced levels of effortful control during middle childhood have been associated with greater likelihood of developing anxiety in later life (Muris et al., 2008). In research examining children with neurodevelopmental conditions, studies have shown that children with ASD tend to have reduced levels of effortful control, as compared with typically developing children and children with developmental delay or Fragile X syndrome, who have relatively higher levels (Bailey et al., 2000; Burrows et al., 2016; Macari et al., 2017). Effortful control has also been associated with internalising problems in children with ASD (De Pauw et al., 2011). Notably, low effortful control has also been identified among infants at elevated likelihood of developing ASD, compared to controls (Clifford et al., 2013; Pijl et al., 2019).

Greater behavioural inhibition and reduced effortful control may therefore represent underlying risk factors for developing internalising-related conditions such as anxiety among infants at elevated likelihood of ASD. While the above studies indicate concurrent associations between temperament, anxiety and ASD, prospective longitudinal cohort studies are needed to establish whether temperament traits precede psychopathology symptoms.

## Parenting-Temperament Associations and the Development of Internalising Disorders

Growing demand for early intervention strategies has motivated a research focus on early environmental factors that may combine with temperamental predispositions to modify trajectories towards affective problems. In particular, parenting has received substantial attention because it is a tractable target for early holistic intervention (Yap et al., 2016). Two parenting variables are especially relevant to interventions focused on attenuating the development of internalising-related symptoms in childhood. Firstly, non-directive parenting, which refers to low levels of intrusive parenting (an overinvolved behavioural style that places demands on the child while limiting autonomy, associated with the development of anxiety; Möller et al., 2016). And, secondly, sensitive parenting, defined as parental responsivity to age-appropriate growth needs in the infant (Feldman et al., 2004), and generally associated with positive socio-emotional child outcomes (Bigelow et al., 2010; Leerkes et al., 2009).

To design effective early interventions, we need to know how parenting interacts with temperament to shape later outcomes. Two statistical approaches facilitate this process: moderation and mediation. Moderation analyses indicate the conditions under which the direction or strength of an effect varies (Holmbeck, 1997); if a predictor variable is related to an outcome variable, but only under certain conditions (‘M’), then M is a moderator variable (Kraemer, 2016). By contrast, mediation analyses can be used to test hypotheses about the mechanism through which a given effect occurs; an independent variable influences the mediator variable which in turn influences the outcome (MacKinnon et al., 2007). This technique allows for the examination of potential causal chains, such as the influence of parenting on child temperament and subsequent developmental outcomes. Identifying moderators and mediators can help investigators better target their early intervention designs; understanding moderating variables may tell us what intervention is most effective for which individuals with what specific difficulty, under which set of circumstances, whilst identifying a mediating path can help investigators more closely target underpinning mechanisms or identify appropriate proxy outcome measures (Breitborde et al., 2010).

### Moderation Relationships

In the general population, parenting behaviours are thought to moderate the relationship between individual differences in infant temperament and the likelihood of developing internalising disorders in later childhood (Ryan & Ollendick, 2018). Nondirective and sensitive parenting behaviours become established early on in the first year of life and remain stable over time (Wan et al., 2013). Several studies have indicated that these dimensions of parenting could have a moderating effect on the relation between infant behavioral inhibition and later affective disorders. For example, Rubin et al. (2002) show that low levels of nondirective parental behaviour increase the likelihood for infants with greater behavioural inhibition to develop symptoms consistent with anxiety. Other studies have shown a similar pattern in mid-to-late childhood, such that the predictive relation between earlier inhibition and later anxiety states is increased by low nondirective parenting (Lewis-Morrarty et al., 2012; Prinzie et al., 2014).

Very little is known about how parental behaviour might moderate the relationship between temperament and internalising/anxiety states in young children at risk of developing neurodevelopmental conditions; however, several studies have identified early differences in nondirective and sensitive parenting in infants with later ASD. Among infants who have an older sibling with ASD, parents of infants who later receive ASD diagnoses show lower nondirectiveness and lower sensitive parenting between 9 and 15 months (Campbell et al., 2015; Srinivasan & Bhat, 2020; Wan et al., 2012). A number of intervention studies have shown that parenting behaviour can also be shifted in elevated-likelihood samples to produce a moderate amelioration in core symptom trajectories (see, e.g., Green et al., 2015, 2017; Ventola et al., 2017) though not in all instances (e.g. Whitehouse et al., 2019); indicating that this is a promising domain to explore in relation to later internalising-related distress within ASD.

### Mediation Relationships

While moderation models can examine how different parenting styles might influence the predictive relation between infant temperament and later psychopathology, mediation models can be used to examine the mechanisms and potential causal chains through which parent behavior shapes and conditions child behaviour and subsequent outcomes (Kasari & Sigman, 1997; Totsika et al., 2011). Mediation models can be useful to unpick the reciprocal transactions between environmental factors and infant characteristics that occur over development (Beauchaine & Hinshaw, 2010; Kiff et al., 2011). One particularly important domain to consider is effortful control, since it has a much longer developmental timecourse than other domains of temperament (Putnam et al., 2001; Rothbart, 1988), has a hierarchical relationship to other domains of function (Nigg, 2017), and is predicted over time by parenting in early childhood (Karreman et al., 2008; Kochanska et al., 2000; Lengua et al., 2007, 2019). Indeed, mediation analyses have shown that, in typically developing populations, greater nondirective parenting associates with higher child resilience through the mediating effect of increased infant effortful control (Taylor et al., 2013). This evidence, combined with the findings from infant sibling cohorts regarding temperamental risk factors for anxiety and ASD, suggest that it may be fruitful to examine whether changes in effortful control mediates any relation between parenting and internalising outcomes in children with a family history of ASD.

## The Present Study

We used a prospective longitudinal design to examine how early parenting behaviour moderates the relation between early-emerging behavioral inhibition and later internalising-related problems; and whether relations between early parental behavior and later child internalising difficulties are mediated by changes in the later-emerging temperament domain of effortful control. Such study designs allow observations of broad phenotypic characteristics expressed in very young relatives of children who have already received a specific developmental disorder diagnosis (Jones et al., 2014; Szatmari et al., 2016). We included infants with an elevated-likelihood of developing ASD (who had an older sibling with ASD) and a typical likelihood control group who were infants with an older sibling with typical development. Our primary models included infant behavioural inhibition and parenting behaviour at 8 and 14 months; toddler effortful control at 24 months, and child internalising behaviour at 36 months. We measured parent-report scores of infant temperament and behaviour, as well as observed parent-infant interaction.

In line with the existing literature, we expected that infant behavioural inhibition would associate with later internalising symptoms. We also hypothesised that: (1) early nondirective parenting would moderate the effects of early infant behavioural inhibition on later child internalising problems; (2) early sensitive parenting behaviour would moderate the effects of early infant behavioural inhibition on later child internalising problems, and (3) the relationship between early nondirective parenting and later reductions in child internalising problems would be mediated by changes in toddler effortful control. Two sets of exploratory analyses were conducted to probe for the influence of: (i) child age and (ii) interactions between child inhibition and effortful control (SM).

## Methods

### Participants

As part of the British Autism Study of Infant Siblings (BASIS; www.basisnetwork.org) 133 infants took part in research assessments at 8, 14, 24 and 36 months (hypotheses 1–2 examined 133 participants while hypothesis 3 examined a subset of 123 participants due to missing data; see Data Analysis plan for more detail). At enrolment, each elevated-likelihood (EL) infant (*N* = 89) had an older sibling with a community clinical ASD diagnosis, confirmed using the Development and Well-Being Assessment (DAWBA; Goodman et al., 2000) and the Social Communication Questionnaire (SCQ; Rutter et al., 2003) by expert clinicians in the team (TC). For further information on the diagnostic status of participants’ older siblings, see SM (Sect. 1).

A control group of 44 infants (which we refer to as TL, due to their typical likelihood of ASD) were full-term infants recruited from a volunteer database at the Birkbeck Centre for Brain and Cognitive Development. At enrolment, all control infants had at least one older sibling with typical development and no first degree relatives with a diagnosis of ASD; the SCQ was used to confirm absence of ASD in older siblings, with no child scoring above instrument cutoff (≥ 15). Ethical approval was obtained from the NHS National Research Ethics Service (NHS RES London REC 08/H0718/76; 14/LO/0170). Parental written consent was obtained at all visits. A subset of the participants described above also participated in a separate randomised control trial that examined a parenting intervention; to ensure the robustness of our results and in the interests of transparency, we address this potentially confounding factor in the Data Analysis plan and Results below.

Exclusion criteria for both groups, based on parent report, included significant prematurity (gestational age ≤ 32 weeks), medical conditions such as epilepsy, heart conditions, vision and hearing impairments, cerebral palsy, and genetic conditions such as Down's syndrome or Fragile X. None of the infants had known any medical or developmental condition at the time of enrolment.

### Measures

#### Infant Temperament

Temperament was captured using the Infant Behaviour Questionnaire-Revised (IBQ-R; Gartstein & Rothbart, 2003) at 8 and 14 months, and the Early Childhood Behaviour Questionnaire (ECBQ; Putnam, Gartstein, & Rothbart, 2006) at 24 months. These parent-report questionnaires ask caregivers to rate the frequency of specific offspring behaviours during the previous two weeks.

Behavioural Inhibition was measured using the IBQ-R Fear subscale at 8 months for our primary analyses (infant distress or an inhibited approach to novel objects, social stimuli or novelty; 13/16 questions on aversive responses to unfamiliar people or places, while 3 probe startle responses to sudden changes). We selected this subscale as a proxy for behavioural inhibition due to the similarity of the questions to Kagan’s original definition (“withdrawal and timidity to the unexpected”; Schmidt et al., 2020, p.7) and given its explicit definition in the IBQ-R as reflecting behaviour denoting ‘inhibition of approach towards novel and/or intense stimuli’ (Gartstein & Rothbart, 2003). Other multi-method approach studies have used IBQ-R Fear (Crockenberg & Leerkes, 2006; Gensthaler et al., 2013) and ECBQ Shyness subscales as a proxy of parent-reported behavioural inhibition to complement observed behavioural inhibition (e.g. Geng et al., 2011) and the fear subscale has been linked to later anxiety in other longitudinal studies (Shephard et al., 2018; Tonnsen et al., 2013). For supplementary models, we additionally used the IBQ-R Fear at 14 months and the ECBQ-Shyness subscale at 24 months (discomfort, slow or inhibited approach to novelty and uncertainty in social situations).

For our primary models, effortful control was measured by the ECBQ (Putnam, Gartstein, & Rothbart, 2006) at 24 months. Effortful control is characterised by the ability of shifting attention, duration of attentional focusing, and low-intensity pleasure. For supplementary analyses we used the related construct of infant regulatory capacity as assessed by the IBQ-R at 14 months (Gartstein & Rothbart, 2003).

#### Parental Sensitivity and Nondirectiveness

The Manchester Assessment of Caregiver-Infant Interaction (MACI; Wan et al., 2016) was used to rate these parenting behaviours based on 6-min parent-infant unstructured play interactions, videotaped at the 8- and 14-month laboratory assessment. The parent was instructed to engage in play as they would do at home, using the set of toys provided if they wished [approximately 96% of parents were mothers; mean age 35.7 years (*SD* = 4.99)]. Clips were later independently rated for the first 6 min from the point the researchers left the room by a trained coder, blinded to participant information, on 7 (7-point) scales. We focused on the two parent scales: nondirectiveness (a low score [i.e. high directive parenting behaviour] represents demanding, intrusive and negative behaviours, and comments directed at the infant not in the service of promoting infant-initiated behaviour) and sensitivity (a high score represents appropriate, contingent, attentive, supportive and immediate responsivity to infant behaviour and developmental need). Excellent psychometric properties and inter-rater reliability were reported in previous studies (Wan et al., 2013, 2016), where ratings were independent of infant gender, infant nonverbal development, parental age and socioeconomic status. Independently blind-rated clips of a proportion of the current sample (26%) showed reasonable to high agreement: single measures intraclass correlations using a two-way mixed effects model (absolute agreement definition) ranged from *r* = 0.68 to 0.83.

#### Internalising Symptoms

At the 36 month visit, the Vineland Adaptive Behaviour Scales, second edition (VABS-II; Sparrow et al., 2005) were completed by parents in an interview, including a subscale that measures internalising symptoms, including anxious and withdrawal-type behaviours. Internalising symptoms measured at approximately 3 years have frequently been shown to relate to later childhood internalising difficulties and affective disorders, and as such internalising score at 36 months was chosen for the outcome variable (Tandon et al., 2009; Whalen et al., 2017). The VABS-II internalising scale comprises 11 items, of which 6 probe anxiety-prone behaviour (e.g. ‘Is overly anxious or nervous’ and ‘Refuses to go to school or nursery because of fear, feelings of rejection or isolation’). Cronbach alpha for the internalising subscale was 0.82 for the current sample. Raw scores in the internalising domain were used in this analyses, as meaningful variation in psychopathological symptoms in non-clinical samples is thought to be obscured by the usual translation of raw scores into standardised scores (Hessl et al., 2009).

#### Developmental Assessment

At each visit, the Mullen Scales of Early Learning (MSEL; Mullen, 1995) were administered to infants to establish a developmental measure based on task performance. Four scales (visual reception, fine motor, receptive and expressive language) were combined to give an early learning composite score [TL = 116.66 (15.02); EL = 105.43 (22.19), 36 months].

#### ASD Diagnosis

Information available from all visits was triangulated by an independent rating team, combined with expert clinical judgement (TC, GP), to determine an ICD-10 (World Health Organization, 2018) ASD classification. Classification was informed by but not dependent on results from the Autism Diagnostic Observation Schedule (ADOS; Lord et al., 1989), a play-based assessment conducted by a trained assessor designed to elicit reciprocal social interaction, language and communication and repetitive stereotyped behaviours.

#### Timepoint Selection

The selection of timepoints for measures of infant temperament and parenting was made on the basis of temporal precedence, which is thought to be theoretically relevant to longitudinal designs (George & Jones, 2000). Given the early emergence of the temperament trait and the stability of the parenting variable, for hypotheses 1–2 behavioural inhibition at 8 months, parenting measures at 14 months, and internalising symptoms at 36 months were selected.

For our third hypotheses, we selected nondirective parenting at 8 months, effortful control at 24 months, and internalising symptoms at 36 months. Effortful control at 24 months was selected given the consensus that this behaviour develops predominantly through the toddler years and upward (Putnam et al., 2001), and as the top-down processes implicated in effortful control are not developed until the second year of life (Hendry et al., 2016; Kochanska et al., 2000).

To explore whether our prespecified hypotheses missed additional information, additional model variants (including different timepoints or switching from mediation to moderation) were examined (Sects. 3, 4, SM). Post-hoc exploratory analyses examining the influence of parental behaviour on infant effortful control where infant behavioural inhibition is also taken into account (Sect. 7, SM).

## Data Analysis

For our primary models, Pearson’s correlation coefficients were calculated to assess the relationships between predictors (temperament traits measured at 8 and 24 months; parent–child interaction domains measured at 8 and 14 months) and internalising measured at 36 months. The reported significance level was set to *p* < 0.05, unless otherwise specified (e.g. due to the high number of comparisons, the significance level for bivariate correlations was set to *p* < 0.01). All predictor and outcome variable correlation coefficients were calculated using SPSS 25. Group differences of temperament and parenting variables were also analysed for our primary models; sample characteristics, including means and standard deviations for measures and risk group comparisons (effect sizes), were calculated.

## Hypotheses 1–2

## Nondirective and Sensitive Parenting as Moderators of Internalising Symptoms

**Fig. 1:**
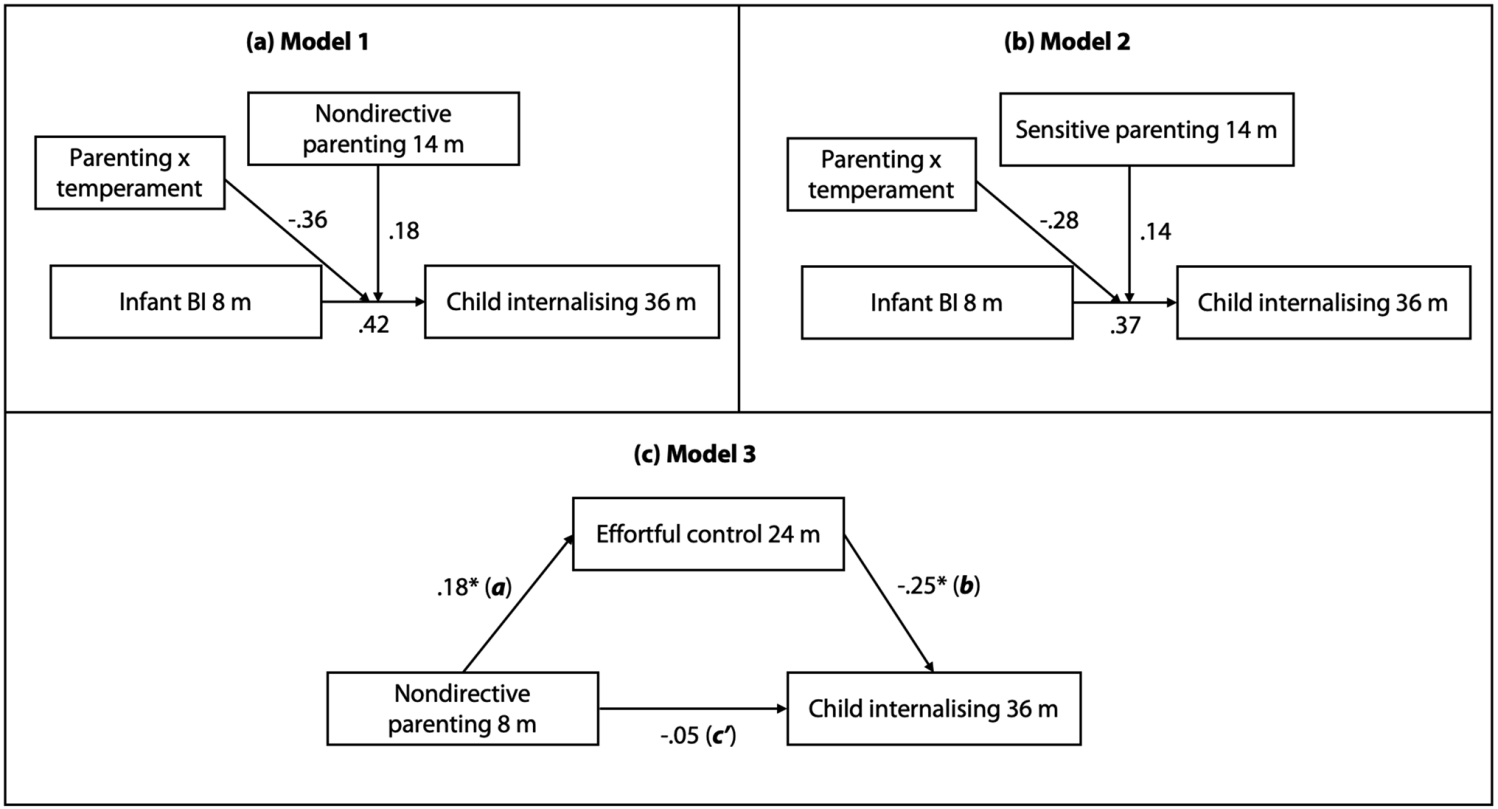
Schematic showing the relationships between variables in the moderation analyses (Hypotheses 1–2) and mediation analysis (Hypothesis 3). Labels a, b and c’ are path coefficients representing standardised coefficients; the c-prime path refers to the direct effect. *p < 0.01

For our first and second hypotheses (Fig. 1), child internalising scores at 36 months were regressed onto inhibited infant temperament at 8 months, as were two interaction terms: nondirective parenting at 14 months x infant inhibition (Hypothesis 1) and sensitive parenting at 14 months × infant inhibition (Hypothesis 2). Grand-mean centred scores were used to compute the interaction terms. We probed statistically significant interactions at one standard deviation below and one standard deviation above the interaction terms (Aiken et al., 1991).

## Hypothesis 3

## Effortful Control as a Mediator of Nondirective Parenting and Internalising Symptoms

In our mediation model (model 3, Fig. 1), we were specifically interested in measuring: (1) the direct paths from nondirective parenting at 8 months to effortful control at 24 months and internalising symptoms at 36 months; (2) the direct paths from effortful control at 24 months to internalising symptoms at 36 months, and (3) indirect paths from nondirective parenting to internalising symptoms via effortful control. Tests of statistical mediation employed bootstrapping with 10000 samples to generate bias-corrected confidence intervals for indirect effects (Shrout & Bolger, 2002).

All analyses testing hypotheses were conducted in Mplus 7.13 (Muthén & Muthén, 1998–2015). Maximum Likelihood Robust (MLR) estimation was used to provide robust standard errors to account for the non-normal distribution and skewness in the internalising measure. All parameter estimates were standardised and thus indicate how much the dependent variables would be expected to change for a single standard deviation change in the predictor variable.

Hypotheses were tested using observed (i.e. non-latent) variables only and were estimated using the full sample (*n* = 133) for hypotheses (1–2) and the subset with available parent–child interaction for hypothesis (3), where we assumed data was missing at random (total *n* = 123). Likelihood group status was treated as a covariate in all models to control for effect and regressed on each predictor and the internalising variable. Group differences in the temperament variables were also tested.

In addition, 17 of the 133 participants in the present sample (12.8%) were assigned an ASD diagnosis at 36 months. To exclude the possibility that main effects were influenced by ASD outcome, we repeated the analyses in each model after omitting participants with a 36-month ASD diagnosis.

Finally, between the first and second timepoints, 22 infants in the elevated-likelihood group participated in the intervention arm of an randomised controlled trial (RCT) of a parent-mediated early intervention programme (Green et al., 2015, 2017). To exclude the possibility of confounding effects, supplementary analyses were conducted (see SM, Sect. 5 for full details).

## Results

### Sample Characteristics

Descriptive statistics of our sample characteristics and our infant and parenting measures are shown in Table 1. The groups at typical likelihood (TL) and elevated likelihood (EL) of developing ASD did not differ in the proportion of girls and were the same age at each visit with the exception of the 24-month timepoint.

**Table 1:** Sample characteristics and descriptives by group. Sample characteristics, means and standard deviations for measures and group comparisons (effect sizes)

	Typical likelihood M (SD)^N^	Elevated likelihood M (SD)^N^	Group differences
Visit 1 (8 months)			
% girls	59.1%^*N* = 44^	52.3%^*N* = 88^	*n/s*
Age in months	7.41 (1.23)^*N* = 44^	7.90 (1.18)^*N* = 88^	*n/s*
Mullen ELC	104.70 (11.60)^*N* = 44^	101.56 (13.94)^*N* = 88^	*n/s*
Behavioural inhibition	2.50 (.94)^*N* = 44^	3.11 (1.15)^*N* = 86^	*F* (1, 129) = 9.25, *p* = 0.003, η^2^ = 0.07
Nondirective parenting	3.98 (1.37)^*N* = 41^	3.00 (1.18)^*N* = 37^	*F* (1, 77) = 11.26, *p* = 0.001, η^2^ = 0.13
Visit 2 (14 months)			
% girls	60.5%^*N* = 43^	(52.8%)^*N* = 89^	*n/s*
Age in months	13.93 (1.28)^*N* = 43^	14.15 (1.23)^*N* = 89^	*n/s*
Mullen ELC	107.60 (15.34)^*N* = 43^	97.83 (15.15)^*N* = 89^	*F* (1, 131) = 11.97, *p* = 0.001, η^2^ = 0.09
Nondirective parenting	4.28 (1.39)^*N* = 43^	3.51 (1.47)^*N* = 41^	*F* (1, 83) = 6.06, *p* = 0.02, η^2^ = 0.07
Sensitive parenting	4.09 (1.48)^*N* = 43^	3.46 (1.44)^*N* = 41^	*F* (1, 83) = 4.16, *p* = 0.04, η^2^ = 0.05
Visit 3 (24 months)			
% girls	62.5%^*N* = 40^	54.2%^*N* = 83^	*n/s*
Age in months	23.90 (0.71)^*N* = 40^	25.42 (1.93)^*N* = 83^	*F* (1, 122) = 23.21, *p* = 0.000, η^2^ = .16
Mullen ELC	116.50 (13.72)^*N* = 40^	100.75 (19.11)^*N* = 83^	*F* (1, 122) = 21.73, *p* = 0.000, η^2^ = .15
ECBQ effortful control	4.75 (0.45)^*N* = 40^	4.44 (.61)^*N* = 77^	*F* (1, 116) = 7.65, *p* = 0.007, η^2^ = .06
Visit 4 (36 months)			
% girls	59.1%^*N* = 44^	53.4%^*N* = 88^	*n/s*
Age in months	38 (2.61)^*N* = 44^	38.48 (1.77)^*N* = 88^	*n/s*
Mullen ELC	116.66 (15.02)^*N* = 44^	105.43 (22.19)^*N* = 88^	*F* (1, 131) = 9.15, *p* = 0.003, η^2^ = 0.07
VABS-II internalising	0.77 (1.08)^*N* = 44^	1.79 (2.34)^*N* = 89^	*F* (1, 131) = 7.01, *p* = 0.007, η^2^ = 0.05

*Mullen ELC* mullen early learning composite, *IBQ* infant behaviour questionnaire, *ECBQ* early childhood behaviour questionnaire, *BI* behavioural inhibition, *EC* effortful control, *VABS-II Internalising* vineland adaptive behaviour scale, second edition—internalising score

Significance threshold set to *p* = 0.01

The EL group scored significantly higher than the typical-likelihood group on the behavioural inhibition scale at 8 months and the effect size was below moderate (η^2^ = 0.07). The EL group had significantly lower effortful control than the TL group at 24 months; the effect size was also below moderate (η^2^ = 0.06). Nondirective parenting was higher in the TL group at 8 and 14 months (all η^2^ ≤ 0.13), as was sensitive parenting at 14 months (all η^2^ ≤ 0.09). Scores on the Mullen Early Learning Composite (Mullen, 1995) were higher in the TL group at 14, 24 and 36 months (all η^2^ ≤ 0.1.5). The EL group scored higher than the TL group on the internalising subscale at 36 months (η^2^ = 0.05).

### Bivariate Correlations

Table 2 shows correlations among the predictor and internalising variables for our primary models in the analysis for the full sample. Higher effortful control at 24 months related to higher nondirective parenting at 8 months, and lower internalising at 36 months. Nondirective parenting was correlated across time points, suggesting stability. Sensitive and nondirective parenting, were inter-correlated. Correlations of key variables at alternative timepoints are recorded in the SM (Table S1, SM).

**Table 2:**
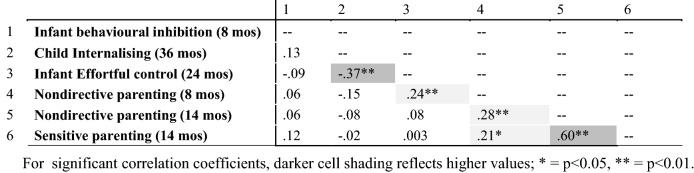
Bivariate correlations for primary model variables. Items 1–3 are parent-report measures; items 4–6 are parent– child interaction observations

### Models 1–2: Nondirective and Sensitive Parenting as Moderators of Internalising Symptoms

In model 1 (see Fig. 1, Table 3), contrary to hypothesis 1, there were no significant associations between infant behavioural inhibition at 8 months or nondirective parenting at 14 months and internalising symptoms at 36 months, either independently (all *p* ≥ 0.16) or interactively (β = − 0.36, *p* = 0.45, 95% CI [− 1.04, 0.54]). This remained unchanged after adjustment for the potentially confounding effects of group status (EL vs. TL). In model 2 (see Fig. 1, Table 3), we assessed whether an interaction between sensitive parenting at 14 months and infant behavioural inhibition at 8 months was associated with internalising symptoms at 36 months, but the results disconfirmed hypothesis 2 that infants who experienced more sensitive parenting would also have lower internalising scores in toddlerhood (β = − 0.28, *p* = 0.49, 95% CI [− 0.98, 0.30]). Additionally, no significant results were found in the model variants in which the same measures taken at different timepoints were entered into the model (Table S2, SM). Excluding children with an ASD diagnosis at 36 months led to no changes to the null findings of the moderation model analyses. Full details are reported in the Supplementary Materials (Table S6).

**Table 3:** Standardised model results of moderation and mediation analyses. Models 1–3 refer to hypotheses 1–3 shown in Fig. 1

	Predicting internalising (36 mos)				
	Predictor	β	*p*	LLCI 95%	ULCI 95%
Model 1					
Infant BI, 8 months		0.42	0.18	− 0.16	0.89
Nondirective parenting, 14 months		0.18	0.54	− 0.33	0.60
Group status		0.20	0.001	0.09	0.29
BI × NDP		− 0.36	0.45	− 1.04	0.54
Model 2					
Infant BI, 8 months		0.37	0.23	− 0.05	0.94
Sensitive parenting, 14 months		0.14	0.55	− 0.23	0.54
Group status		0.20	0.001	0.10	0.29
BI × SP		− 0.28	0.49	− 0.98	0.30

	Predictor	Mediator	Total effect (SE)	Direct effect (SE)	Indirect effect (95% CI bootstrap)
Model 3					
Nondirective parenting, 8 months		Effortful control, 24 months	− 0.09 (0.09)	− 0.05 (0.09)	− 0*.*05 (− 0.11, − 0.01)

*BI* behavioural inhibition, *NDP* nondirective parenting, *Group status* membership of the typical likelihood or elevated likelihood group, *SP* sensitive parenting, *BI × NDP* interaction term, behavioural inhibition × non-directive parenting, *BI × SP* interaction term, behavioural inhibition × sensitive parenting, *LLCI* lower limit confidence interval, *ULCL* upper limit confidence interval, *CI* confidence interval. In model 3, group status was entered as a covariate

*p ≤ 0.05

### Model 3: Effortful Control as a Mediator of Nondirective Parenting and Internalising Symptoms

In model 3, tests of direct effects demonstrated that nondirective parenting at 8 months was positively associated with effortful control at 24 months (β = 0.18, *SE* = 0.09, *p* = 0.04). The model also demonstrated that effortful control at 24 months inversely associated with internalising symptoms at 36 months (β = − 0.25, *SE* = 0.09, *p* = 0.006). There was no significant direct effect present between nondirective parenting and internalising symptoms (β = − 0.05, *SE* = 0.09, *p* = 0.62). Group status was significantly associated with effortful control at 24 months (β = − 0.22, *SE* = 0.08, *p* = 0.007) and internalising scores at 36 months (β = 0.15, *SE* = 0.06, *p* = 0.02). Predictor variables can exert an indirect effect on an outcome variable through a mediating variable in the absence of an association between predictor and outcome variable (given that a total effect is the sum of many different paths of influence, direct and indirect, not all of which may be part of the formal model; Hayes, 2009). As such we proceeded to investigate indirect effects of nondirective parenting to internalising symptoms through effortful control. Results from tests of indirect effects indicated that, consistent with our hypothesis, effortful control at 24 months lies on the path between nondirective parenting at 8 months and internalising symptoms at 36 months when controlling for group status (β = − 0.05, 95% CI BS [− 0.11, − 0.01]). Direct and indirect effects are shown in Fig. 1 and Table 3, respectively.

A variant of model 3 conducted testing effortful control at 14 months rather than 24 months (model 3.2, Table S3, SM) was found to be non-significant. A model variant including parenting measured at 14 months rather than 8 months was also conducted and found to be non-significant (model 3.3, Table S3, SM). A post-hoc exploratory moderated mediation analyses tested the extent to which nondirective parenting predicting child internalising through effortful control varied contingent on the level of behavioural inhibition (Fig S1, SM). The effect was significant, suggesting that higher levels of behavioural inhibition would make infants less susceptible to the effects of parenting on effortful control (Table S7, SM).

To assess whether diagnosis of ASD at 36 months influenced the main effects, we also repeated the original analyses adding diagnostic outcome as a binary variable, representing diagnosis of ASD at 36 months. Excluding children with an ASD diagnosis at 36 months in the mediation model led to no indirect effect (β = − 0.03, 95% CI BS [− 0.09, 0.02]). Full details are reported in the Supplementary Materials (see Sect. 6). Control analyses of the influence of participants’ inclusion in an RCT resulted in null findings, suggesting the absence of confounding effects in this regard (see Sect. 5 in SM).

## Discussion

Three hypotheses were tested to understand the role of temperament in the relationship between early parenting behaviour and internalising problems within ASD. Evidence supported our third hypothesis in our enriched-ASD sample: more nondirective parenting behaviour in the first year of life was related to less child internalising at three years via the mediating variable of effortful control in toddlerhood. No direct link was found between nondirective parenting behaviour and child internalising, thus highlighting the mediating role of effortful control in toddlerhood which develops with parental support. However, it is notable that the main effect resulting from tests of our third hypothesis disappeared once children with an ASD diagnosis were removed from the model. While this difference may be explained by a reduction in statistical power, it could suggest that the diagnosed children were driving the effect. No support was found for our two other hypotheses regarding the moderating impact of either more nondirective parenting behaviour or more sensitive parenting behaviour at 14 months on the relationship between behavioural inhibition at 8 months and internalising problems at 36 months.

### Behavioural Inhibition (Hypotheses 1 and 2)

Early behavioural inhibition predicts internalising problems later in life in typically developing populations (Clauss & Blackford, 2012; Kostyrka-Allchorne et al., 2020; Muris et al., 2011) as well as those at elevated likelihood of developing ASD (Ersoy et al., 2020; Shephard et al., 2018). Although we found a bivariate correlation between behavioural inhibition at 14 months and internalising scores at 36 months (Table S1, SM), our findings give no indication that nondirective nor sensitive parenting would act to mitigate or alter the path from early behavioural inhibition to child internalising problems in an ASD-enriched cohort. This null finding corroborates several studies that suggest no risk-enhancing effects of traditionally negative parenting behaviours, such as parental overprotection, when interacting with infant temperament in typically developing populations (Sentse et al., 2009; Vreeke et al., 2013). An exception to this pattern is Rubin et al. (2002) finding that intrusive parenting behavior significantly moderated the relationship between toddler inhibition and preschool social reticence; this discrepancy may be explained by different assessment methodologies (i.e. the use of behavioural paradigms to measure inhibition as opposed to parent-report) as well as sample characteristics.

Based on our null findings and the pattern of our bivariate correlations, as well as the previous literature, evidence suggests sensitive parenting behaviour does not seem to act as a protective factor for children who show high behavioural inhibition, and the relative tendency toward low nondirective behaviour observed in parents of infants at elevated likelihood of ASD (Wan et al., 2013) does not interact with behavioural inhibition to explain internalising behaviour at 36 months.

### Effortful Control (Hypothesis 3)

Recent research has implicated a role of low effortful control in the development of internalising-related distress in young children with (Ersoy et al., 2020) and without (White et al., 2011) ASD. Our results extend on this by identifying, for the first time, the role of effortful control in elucidating the link between parenting behaviour and internalising symptoms in an ASD-enriched cohort. Although previous research has found evidence for the contribution of parenting behaviour and behavioural inhibition to later internalising symptoms (Ryan & Ollendick, 2018), the pathway from nondirective parenting to internalising behaviour via effortful control in this population is novel.

Our post-hoc moderated mediation analyses also indicated that nondirective parenting had a greater effect on effortful control (and subsequent internalising symptoms) when infants have less behavioural inhibition. This exploratory analysis suggests a potential alternative risk path, whereby children low in behavioural inhibition are more sensitive to the protective factor of nondirective parenting, but children high in behavioural inhibition are less so. This potential differential susceptibility to parenting behaviour, based on infant temperament, may be important to conceptualise when investigating parent-mediated risk for the development of internalising-related distress in such cohorts in the future. Indeed, an alternative approach to the present study would be to test temperamental moderators of the relationship between early parenting and later child adjustment outcomes. These relationships can be studied within two relevant frameworks: the goodness-of-fit concept (proposing a match between parental behaviour and child temperament gives rise to optimal development, whereas a mismatch leads to suboptimal functioning; Thomas & Chess, 1977), and differential susceptibility theory (proposing that certain children have greater sensitivity to supportive and stressful environments, ‘for better and for worse’; Belsky et al., 2007). Such an approach would facilitate study of the effects of ‘type of child’ on the relationship between parenting and anxiety within ASD, representing a possible direction for future research.

One question raised by our findings, relating to differential susceptibility, is why the mediation effect in Hypothesis 3 was no longer significant once infants who went on to develop an ASD diagnosis were removed from the model. This may simply be explained by reduced statistical power. An alternative explanation could be that these children (whose parents score low on nondirective behaviour on average) may be more susceptible to the effects of parenting on their levels of effortful control, and may subsequently be more likely to develop internalising-related distress. This raises an important possibility for a parent-mediated intervention targeting effortful control in this group.

### Clinical and Theoretical Implications

Exploring the relationship between parental behaviour and infant temperament factors may be fruitful for understanding parent-mediated risk for psychopathology in ASD-enriched cohorts. Future studies focusing on the potential for parenting behaviour to support the development of infant effortful control may provide further evidence for parent-mediated interventions. Parenting may be a suitable intervention target for several reasons. Firstly, parent-mediated interventions within ASD-enriched cohorts have already been successful in increasing parental nondirectiveness by enriching parenting sensitivity and increasing parental awareness of the importance of their own behaviours in relation to the infant, which may be particularly relevant when an infant is displaying communicative cues that are more subtle than usual (Green et al., 2015, 2017). Secondly, less nondirective behaviour in parents may reflect parental stress or mood problems (Möller et al., 2015), both of which are likely to be heightened in the postnatal period; parents may use a less nondirective approach if they are unsure how to be effective in their parenting behaviour, or if they are otherwise low in emotional availability (for example, if caught up with financial or relational stress). If nondirective parental behaviour facilitates the development of child self-regulatory skills by giving the child more time and opportunity to use these skills without intrusion, then interventions incorporating components such as sensitivity training and increased social support may help increase parental nondirectiveness and subsequent infant effortful control. These questions represent a promising avenue for further study.

The current findings also add to the broader evidence base for the potential role of effortful control as a protective or compound risk factor in child development (e.g., Taylor et al., 2013). Low effortful control in infancy is commonly seen in children who go on to have ASD and ADHD (Johnson et al., 2015) or internalising difficulties (Kostyrka-Allchorne et al., 2019). It has been suggested that high levels of effortful control may compensate for a range of different atypicalities early in life, explaining why infants with high effortful control are less likely to receive any diagnosis later in childhood (Johnson, 2012). Higher levels of effortful control in early childhood also correlate with a range of socio-economic and health outcomes in adulthood, even when controlling for intelligence, social class and shared family background (Moffitt et al., 2011). While our moderated mediation findings suggest that effortful control confers more benefits in the context of lower behavioural inhibition, indicating that effortful control may be more or less protective contingent on other temperamental dimensions, taken together the evidence suggests that effortful control could be a useful target for intervention, given increased effortful control has positive benefits in a range of cases (though see Henderson et al., 2015).

While our findings suggest effortful control represents a potentially ‘malleable’ factor that may modify developmental trajectories, behavioural inhibition may instead represent a more ‘fixed’ risk for later psychopathology. The age of emergence of behavioural inhibition is thought to be from 4 months, with physiological antecedents detectable earlier on (Pérez-Edgar & Fox, 2018). Inhibited social behaviour shows longitudinal stability from the first year of life to early and middle childhood, as well as into adolescence (e.g. Brooker et al., 2016; Calkins et al., 1996; Pérez-Edgar et al., 2010). By contrast, it is difficult to measure effortful control before the second year of infancy. Top-down effortful processes required for executive function are not sufficiently developed in the early developmental stages (Kochanska et al., 2000), consistent with the fact that we found a weaker relationship when we substituted effortful control at 14 months into model 3, than when it was originally conducted with effortful control at 24 months (compare Table 3 and Table S3, SM). This developmental timing may make the processes associated with effortful control more susceptible to environmental input than inhibitory processes. The potential, relative fixedness of behavioural inhibition compared with the malleability of effortful control suggests that these two temperamental factors could act separately on later psychopathology risk, representing two distinct paths.

As in the wider literature (Pérez-Edgar & Fox, 2018; Rubin et al., 2002), our findings show that higher levels of infant behavioural inhibition relate to high levels of child internalising. We also show that low levels of nondirective parenting relate to later reduced child internalising, through effortful control, and that this relationship may be stronger in the context of low behavioural inhibition. However, our findings suggest that the predictive value of behavioural inhibition on later internalising is not altered by or dependent on nondirective parental interactions. This counterintuitive finding could be explained by age specificity. In model 3, we show that our mediation analyses are significant when parenting is measured at 8 months—but this significance disappears when parenting is measured at 14 months (model 3.3, Table S3, SM). Our moderation analyses remain unchanged when we adjust for age (Table S2, SM), but this may be because there is no direct relationship between nondirective parenting and later child internalising; without effortful control in the model, differences are undetectable (though see null results in model 2.5, S2, SM).

### Limitations

Findings from this study should be considered in light of several limitations. While we found support for parental nondirectiveness measured at 8 months associating with decreasing internalising problems via 24-month effortful control, it was not possible to disentangle whether nondirective parental interaction impacts on effortful control or whether this behaviour in the parent emerges as a consequence of early emerging signs of effortful control in the child (e.g. early compliance that allows the parent to avoid giving too much direction). In addition, while parental nondirectiveness was measured from observational data, temperament and internalising measures were based on parent-report, which (as well as being potentially susceptible to shared method variance effects; Podsakoff et al., 2012) may be affected by parent psychopathology.

Finally, the generalisability of the present study of infant siblings may be limited in two ways: (i) generalisation to the broader population of children with ASD, but without a sibling with the condition, may be limited since having a first-degree relative with ASD may have influenced sampling of families, and long-term monitoring and evaluation of the development of the infant sibling might have influenced their developmental trajectory (Szatmari et al., 2016); (ii) generalisation to typically-developing children may be limited since the modest sample size of the TL group in this study prohibited us from examining multi-group models, which would indicate whether the findings were consistent, and therefore likely generalisable, for both EL and TL groups.

### General Conclusion

Our data show that effortful control, itself influenced by nondirective parenting behaviour, can act as an ameliorating influence on the path to internalising-related distress within ASD-enriched cohorts; nondirective parenting behaviour may impact on effortful control in toddlerhood. Studies using more specific anxiety measures, as well as multi-method methodologies to examine multidirectional relations between parenting and infant temperament (including subcomponents of effortful control, representing different attentional processes) may be promising steps on the path to informing early intervention approaches.

## Supplementary Information

Below is the link to the electronic supplementary material.

Supplementary file (DOCX 64 kb)
