# Supplementary material for: Infant Effortful Control Mediates Relations Between Nondirective Parenting and Internalising-Related Child Behaviours in an Autism-Enriched Infant Cohort
Source: J Autism Dev Disord. 2021 Aug 26;52(8):3496–511. doi: 10.1007/s10803-021-05219-x (PMC9296408; doi:10.1007/s10803-021-05219-x)
Supplement: Supplementary file 1 — Supplementary file (DOCX 64 kb) [file 10803_2021_5219_MOESM1_ESM.docx]

**Supplementary Materials: Infant effortful control mediates relations between nondirective parenting and internalising-related child behaviours in an autism-enriched infant cohort**

Table of Contents

[1. Sample characteristics 2](#_Toc63337320)

[Diagnostic status of participants’ older siblings 2](#_Toc63337321)

[2. Bivariate correlations 2](#_Toc63337322)

[3. Moderation model variants 2](#_Toc63337323)

[4. Mediation model variants 3](#_Toc63337324)

[5. Intervention trial supplementary analyses 4](#_Toc63337325)

[6. Effects of ASD outcome 5](#_Toc63337326)

[7. Moderated Mediation 5](#_Toc63337327)

[8. References 6](#_Toc63337329)

# Sample characteristics

## Diagnostic status of participants’ older siblings

For all 89 children with an older sibling with a community clinical diagnosis of ASD (hereafter probands) parents completed the Development and Well-Being Assessment (DAWBA: Goodman, Ford, Richards, Gatward, & Meltzer, 2000) and/or the Social Communication Questionnaire (SCQ: Rutter, Bailey, & Lord, 2003). Sixty-seven probands met criteria on both the DAWBA and SCQ. Eight children scored below threshold on the SCQ and two were missing the SCQ, but no exclusions were made due to meeting threshold on the DAWBA and expert opinion. For 12 probands, confirmation of local clinical diagnosis was only available via the SCQ. Screening for possible ASD in the older siblings of the typical likelihood (TL) infants was undertaken using the SCQ, with no child scoring above the instrument cut-off for ASD (>15). For one TL child the SCQ was missing. Medical history review confirmed a lack of ASD within first-degree relatives.

# Bivariate correlations

*Table S1*

|  |  | 1 | 2 | 3 | 4 | 5 | 6 | 7 | 8 | 9 | 10 |
| --- | --- | --- | --- | --- | --- | --- | --- | --- | --- | --- | --- |
| **1** | **Infant BI (8 m)** | -- | -- | -- | -- | -- | -- | -- | -- | -- | -- |
| **2** | **Infant BI (14 m)** | .56** | -- | -- | -- | -- | -- | -- | -- | -- | -- |
| **3** | **Child internalising (3 yrs)** | .13 | .25** | -- | -- | -- | -- | -- | -- | -- | -- |
| **4** | **Infant RC (14 m)** | -.10 | -.18** | -.33** | -- | -- | -- | -- | -- | -- | -- |
| **5** | **Infant EC (24 m)** | -.09 | -.19** | -.37** | .54** | -- | -- | -- | -- | -- | -- |
| **6** | **Nondirective parenting (8 m)** | .06 | -.06 | -.15 | .19* | .24** | -- | -- | -- | -- | -- |
| **7** | **Nondirective parenting (14 m)** | .06 | .11 | -.08 | -.06 | .08 | .28** | -- | -- | -- | -- |
| **8** | **Sensitive parenting (8 m)** | .07 | .05 | -.09 | .06 | .18* | .61** | .29** | -- | -- | -- |
| **9** | **Sensitive parenting (14 m)** | .12 | -.01 | -.02 | -.01 | .003 | .21* | .60** | .40** | -- | -- |
| **10** | **Group status** | .17** | .21** | .25** | -.22** | -.21** | -.19* | -.14 | -.11 | -.01 | -- |

Table S1: Bivariate Correlations for all model variables. Items 1-5 are parent-report measures; items 6-11 are parent-child interaction observations; group status indicates likelihood of developing ASD based on the presence of a diagnosis amongst a first degree relative (two groups: Typical-likelihood or Elevated-likelihood). BI – behavioural inhibition; RC – regulatory capacity; EC – effortful control. * = p<.05, ** = p<.01.

# Moderation model variants

Our first hypothesis predicted that non-directive parenting at 14 months would moderate the relationship between infant behavioural inhibition and internalising at 36 months; our second hypothesis predicted that sensitive parenting at 14 months would moderate the relationship between infant behavioural inhibition at 8 months and internalising at 36 months. Results from tests of these hypotheses were all found to be non-significant. To test for consistency, post-hoc analyses were also conducted to investigate whether the moderating relationship remained non-significant if the same variables measured at different timepoints were entered into the model. For reader interest, we also probed the role of effortful control as a moderator of the relationship between nondirective parenting and later internalising. Results of the tests of moderation were all non-significant, even when multiple timepoints were tests in different configurations (see Table S2).

*Table S2*

|  | *Predicting Internalising (36 mos)* |  |  |  |  |
| --- | --- | --- | --- | --- | --- |
|  | Predictor | β | *p* | LLCI 95% | ULCI 95% |
| Model 1.2 | Infant BI, 14 months | .17 | .60 | -.70 | .92 |
|  | Nondirective Parenting, 8 months | -.29 | .39 | -1.2 | .48 |
|  | BI*NDP | .18 | .71 | -.87 | 1.7 |
| Model 1.3 | Infant BI, 8 months | .45 | .14 | -.40 | 1.1 |
|  | Nondirective Parenting, 8 months | .05 | .85 | -.73 | .65 |
|  | BI*NDP | -.31 | .43 | -1.2 | .96 |
| Model 1.4 | Infant BI, 14 months | .48 | .14 | -.42 | 1.3 |
|  | Nondirective Parenting, 14 months | .16 | .66 | -.73 | 1.1 |
|  | BI*NDP | -.39 | .48 | -1.7 | 1.1 |
| Model 2.2 | Infant BI, 14 months | .36 | .33 | -.64 | 1.2 |
|  | Sensitive Parenting, 8 months | -.01 | .99 | -.85 | .79 |
|  | BI*SP | -.14 | .78 | -1.2 | 1.2 |
| Model 2.3 | Infant BI, 8 months | .51 | .12 | -.34 | 1.2 |
|  | Sensitive Parenting, 8 months | .11 | .60 | -.48 | .64 |
|  | BI*SP | -.38 | .31 | -1.2 | .63 |
| Model 2.4 | Infant BI, 14 months | .28 | .33 | -.46 | 1.1 |
|  | Sensitive Parenting, 14 months | .05 | .87 | -.77 | .87 |
|  | BI*SP | -.08 | .86 | -1.2 | 1.1 |
| Model 2.5 | Nondirective parenting, 8 months | -.37 | .71 | -1.96 | 1.34 |
|  | Effortful control, 24 months | -.37 | .19 | -.83 | .10 |
|  | NDP*Effortful control | .34 | .75 | -1.41 | 2.03 |
| Model 2.6 | Sensitive parenting, 8 months | -.24 | .74 | -2.9 | 2.06 |
|  | Effortful control, 24 months | -.40 | .15 | -.3.4 | .51 |
|  | SP*Effortful control | .23 | .77 | -.46 | .62 |
| Table S2: Standardised model results of exploratory moderation analyses (model variants 1-2). Models 1.2-1.4 and 2.2-2.6 are variants of hypotheses 1 and 2 respectively, replicated with all available timepoints (see Figure 1 in main text); BI - behavioural inhibition; NDP – non-directive parenting; SP – sensitive parenting; BI*NDP – interaction term, behavioural inhibition x non-directive parenting; BI*SP – interaction term, behavioural inhibition x sensitive parenting; SP*Effortful Control – interaction term, sensitive parenting x effortful control; LLCI – lower limit confidence interval; ULCL – upper limit confidence interval; *p ≤ .05. | | | | | |

# Mediation model variants

Our third hypothesis predicted that nondirective parenting would lead to later infant effortful control and subsequent reductions in internalising problems. In our main model, we measured nondirective parenting at 8 months, effortful control at 24 months and internalising score at 36 months, on the basis that mediation models of temporal precedence, which is thought to be theoretically relevant to longitudinal designs (George & Jones, 2000). To test for consistency, post-hoc analyses were also conducted to investigate whether the mediating relationship remained significant if the same variables measured at different timepoints were entered into the model. Nondirective parenting at 8 months, effortful control at 14 months and internalising score at 36 months was tested (model 3.2), as was nondirective parenting at 14 months, effortful control at 24 months and internalising score at 36 months (3.3). Model 3.2 was not significant (β=-.08, 95% CI BS [-.16, .02]), neither was Model 3.3 (β=-.03, 95% CI BS [-.10, .01]). For reader interest, we also probed the role of sensitive parenting as a predictor variable, as well as behavioural inhibition/Shyness as a mediator variable. See Table S3 below.

*Table S3*

| **Model** | **Predictor** | **Mediator** | **Outcome** | **Total Effect (SE)** | **Direct Effect (SE)** | **Indirect Effect**  **(95% CI Bootstrap)** |
| --- | --- | --- | --- | --- | --- | --- |
| 3.2 | NDP, 8m | Infant regulatory capacity, 14m | Internalising, 36m | -.15 (.08)^†^ | -.07 (.08) | -.08 (-.16, .02) |
| 3.3 | NDP, 14m | Effortful control, 24m | Internalising, 36m | -.004 (.09) | .03 (.08) | -.03 (-.10, .01) |
| 3.4 | NDP, 8m | Shyness, 24m | Internalising, 36m | -.21 (.14) | -.21 (.13) | -.003 (-.12, .11) |
| 3.5 | SP, 14m | Effortful control, 24m | Internalising, 36m | .04 (.12) | .04 (.12) | -.002 (-.08, .07) |

Table S3: Standardised model results of exploratory mediation analyses. NDP – non-directive parenting. SP – sensitive parenting. *p≤.05; ^†^p≤06.

# Intervention trial supplementary analyses

Forty-three participants in the elevated-likelihoood (EL) group in this sample took part in a randomized controlled trial (RCT) of a parent-mediated early intervention programme (Green et al., 2015, 2017). The intervention period was between the 8 and 14 month visits. Twenty-two children were in the intervention arm of the trial. To investigate whether enrolment in the RCT or receiving the intervention affected the outcomes described above, we conducted multiple regressions with binary variables representing participation in the RCT and receiving treatment as control variables. The results showed no significant effects of moderation in models 1-2, and this remained unchanged after adjustment for the potentially confounding effects of RCT participation or intervention receipt (see Table S4). Similarly, the finding of an indirect effect within model 3 remained unchanged after analysing the above covariates (see Table S5).

|  | *Predicting Internalising (36 mos)* |  |  |  |  |
| --- | --- | --- | --- | --- | --- |
|  | Predictor | β | *p* | LLCI 95% | ULCI 95% |
| Model 1 | Infant BI, 8 months | .50 | .16 | -.15 | 1.03 |
|  | Nondirective Parenting, 14 months | .20 | .32 | -.35 | .70 |
|  | RCT treatment receipt | -.01 | .86 | -.15 | .13 |
|  | RCT participation | .01 | .86 | -.14 | .12 |
|  | BI*NDP | -.42 | .42 | -.40 | .11 |
| Model 2 | Infant BI, 8 months | .47 | .14 | .03 | 1.05 |
|  | Sensitive Parenting, 14 months | .21 | .42 | -.20 | .62 |
|  | RCT treatment receipt | -.05 | .59 | -.20 | .11 |
|  | RCT participation | .04 | .68 | -.11 | .22 |
|  | BI*SP | -.39 | .35 | -1.12 | .22 |
| Standardised moderation model analyses with covariates. Models 1-2 refer to hypotheses 1-2 shown in Figure 1; BI - behavioural inhibition; NDP – non-directive parenting; SP – sensitive parenting; BI*NDP – interaction term, behavioural inhibition x non-directive parenting; BI*SP – interaction term, behavioural inhibition x sensitive parenting; LLCI – lower limit confidence interval; ULCL – upper limit confidence interval. *p ≤ .05. | | | | | |

*Table S4*

*Table S5*

|  | **Predictor** | **Covariate** | **Mediator** | **Outcome** | **Total Effect (SE)** | **Direct Effect (SE)** | **Indirect Effect (95% CI Bootstrap)** |
| --- | --- | --- | --- | --- | --- | --- | --- |
| Model 3 | NDP, 8m | RCT treatment receipt | Effortful control, 24m | Internalising, 36m | -.21 (.14) | -.09 (.13) | -.08 (-.15, -.02) |
|  | NDP, 8m | RCT participation | Effortful control, 24m | Internalising, 36m | -.24 (.14) | .11 (.14) | -.08 (-.16, -.02) |
| Table S5: Standardised model results of mediation analyses with covariates Model 3 refers to hypothesis (3); NDP– non-directive parenting. *p≤.05. | | | | | | | |

# Effects of ASD outcome

17 children in the EL group were diagnosed with ASD at 3-years; removing children with ASD from the mediation analysis (model 3) changed the main finding such that there was no longer evidence of an indirect effect (β=-.03, 95% CI BS [-.09, .02]). Table S6 shows the effect of removing children diagnosed with ASD from the moderation models, which led to no changes to the existing (null) findings.

*Table S6*

|  | *Predicting Internalising (36 mos)* |  |  |  |  |
| --- | --- | --- | --- | --- | --- |
|  | Predictor | β | *p* | LLCI 95% | ULCI 95% |
| Model 1 | Infant BI, 8 months | .15 | .69 | -.42 | .81 |
|  | Nondirective Parenting, 14 months | -.08 | .80 | -.57 | .47 |
|  | BI*NDP | .10 | .86 | -.86 | .97 |
| Model 2 | Infant BI, 8 months | .40 | .19 | -.04 | .94 |
|  | Sensitive Parenting, 14 months | .14 | .59 | -.26 | .58 |
|  | BI*SP | -.25 | .53 | -.95 | .34 |
| Table S6: Standardised model results of moderation analyses with children with ASD diagnoses excluded. Models 1-2 refer to hypotheses 1-2 shown in Figure 1; BI - behavioural inhibition; NDP – non-directive parenting; SP – sensitive parenting; BI*NDP – interaction term, behavioural inhibition x non-directive parenting; BI*SP – interaction term, behavioural inhibition x sensitive parenting; LLCI – lower limit confidence interval; ULCL – upper limit confidence interval. *p ≤ .05. | | | | | |

# Moderated Mediation

*Figure S1*

-.08 (.03) **

Behavioural inhibition, 14m (Moderator)

-.36 (.11) ***

Internalising, 36m (DV)

-.80 (.38) *

.58 (.48)

Non-directive parenting, 8m (IV)

Effortful Control, 24m (Mediator)

Figure S1: Testing for moderated mediation in the relationships between non-directive parenting and child internalising scores (with behavioural inhibition as the moderator and effortful control as the mediator). Results of moderated mediation analyses: non-directive parenting at 8 months predicts effortful control at 24 months, with the effects moderated by behavioural inhibition. Note: *p < .05, **p < .02, ***p < .002 Solid lines represent significant effects.

## *Table S7*

|  | **Internalising, 36m (SE)** | **Effortful control, 24m (SE)** |
| --- | --- | --- |
| Non-directive parenting, 8m | -.58 (.48) | .36 (.11) *** |
| Behavioural inhibition, 14m | -.01 (.46) | .12 (.11) |
| Non-directive parenting, 8m X behavioural inhibition, 14m | .14 (.14) | -.08 (.03) ** |
| Effortful control, 24m | -.80 (.38) * |  |
| Table S7: Testing for moderated mediation in the relationships between non-directive parenting and child internalising scores (with behavioural inhibition as the moderator and effortful control as the mediator). **p<.05, **p<.02, ***p<.002.* | | |

# References

1. George, J. M., & Jones, G. R. (2000). The Role of Time in Theory and Theory Building. *Journal of Management*, *26*(4), 657–684.
2. Goodman, R., Ford, T., Richards, H., Gatward, R., & Meltzer, H. (2000). The Development and Well-Being Assessment: description and initial validation of an integrated assessment of child and adolescent psychopathology. *Journal of Child Psychology & Psychiatry, 41*(5), 645-655.
3. Rutter, M., Bailey, A., & Lord, C. (2003). *The Social Communication Questionnaire*. Los Angeles, CA: Western Psychological Services
